# Takayasu Arteritis Presenting With Hypotension and Chronic Cerebral Infarctions in a Young Woman: A Case Report

**DOI:** 10.7759/cureus.116992

**Published:** 2026-09-27

**Authors:** Ram Prajapati, Mina Zaki, Kyaw Wanna Win, Abraham Varghese

**Affiliations:** 1 General Medicine, County Durham and Darlington NHS Foundation Trust, Darlington, GBR

**Keywords:** carotid artery stenosis, chronic cerebral infarction, chronic dizziness, large-vessel vasculitis, takayasu arteritis (tak)

## Abstract

Takayasu arteritis (TAK) is a rare large-vessel vasculitis that predominantly affects young women and can present with nonspecific symptoms, making early diagnosis challenging. Neurological manifestations may occur secondary to involvement of the carotid and vertebral arteries and can result in cerebral ischemia. We report the case of a 28-year-old woman who presented with recurrent dizziness, presyncope, palpitations, visual disturbances, and persistent hypotension. Initial neuroimaging demonstrated indeterminate supratentorial hypodensities, while subsequent magnetic resonance imaging of the brain revealed multiple chronic cerebral infarcts involving the bilateral centrum semiovale and an abnormal flow void of the right internal carotid artery. Further computed tomography angiography demonstrated extensive large-vessel disease, including marked narrowing and wall thickening of the bilateral common carotid and subclavian arteries, critical stenosis of the right internal carotid artery, and narrowing of both vertebral arteries. Clinical examination revealed absent peripheral pulses and carotid and subclavian bruits, raising suspicion for large-vessel vasculitis. Following multidisciplinary assessment, the combination of the patient's age, chronic cerebral ischemic changes, extensive arterial disease, pulse deficits, and vascular bruits was considered consistent with TAK. She was treated with intravenous methylprednisolone followed by oral prednisolone and aspirin, with significant improvement in her presenting symptoms.

This case highlights the importance of considering TAK in young patients presenting with unexplained neurological symptoms, hypotension, or chronic cerebral ischemic lesions, particularly when vascular examination reveals pulse deficits or arterial bruits. It also emphasizes the value of comprehensive vascular imaging and multidisciplinary assessment in identifying extensive large-vessel involvement.

## Introduction

Takayasu arteritis (TAK) is a chronic granulomatous large-vessel vasculitis predominantly affecting the aorta and its major branches. The disease may cause arterial wall inflammation, thickening, stenosis, occlusion, and aneurysmal changes, with clinical manifestations determined by the vascular territories involved [1]. The subclavian and common carotid arteries are among the commonly affected branches, while the renal, mesenteric, and vertebral arteries may also be involved [1,2]. TAK predominantly affects women and typically presents before 40 years of age [1]. It is a rare disease worldwide, with reported incidence varying geographically and generally higher rates described in Asian populations than in Europe and North America [2].

Neurological manifestations occur when the carotid or vertebral circulation is affected and may result from cerebral hypoperfusion or thromboembolic mechanisms. Clinical manifestations can include headache, dizziness, visual disturbance, transient ischemic attacks, syncope, and established cerebral ischemia [3]. Because these symptoms may be nonspecific and the neurological examination may remain normal, recognition of the underlying vascular disorder can be challenging [3].

The diagnosis of TAK is based on the integration of clinical findings, laboratory investigations, and vascular imaging. Inflammatory markers may support the diagnosis but are nonspecific and do not necessarily reflect the severity of established arterial damage. The 2022 American College of Rheumatology/European Alliance of Associations for Rheumatology (ACR/EULAR) classification criteria incorporate age at diagnosis, angiographic evidence of aortic or major branch involvement, and clinical features including arterial bruits, reduced pulses, limb claudication, and blood pressure differences between the arms [1]. Vascular imaging is central to diagnosis and assessment of disease extent, with magnetic resonance imaging, computed tomography angiography (CTA), and other vascular imaging modalities providing complementary information [1,4].

We report the case of a young woman presenting predominantly with dizziness, presyncope, visual symptoms, and hypotension, in whom brain imaging demonstrated chronic cerebral infarction and subsequent vascular imaging revealed extensive multiterritorial arterial disease with critical right internal carotid artery stenosis. The case highlights how nonspecific symptoms may represent clinically significant vascular narrowing and emphasizes the importance of careful vascular examination and comprehensive vascular imaging in young patients with unexplained neurological symptoms.

## Case presentation

A 28-year-old woman presented to the emergency department with dizziness, palpitations, and left-sided chest pain. She had experienced intermittent symptoms for several months, initially consisting of headaches, dizziness, visual disturbances, and reduced energy. She had previously been diagnosed with migraine and prescribed propranolol 10 mg twice daily; however, this did not improve her symptoms and was subsequently discontinued because of dizziness and low blood pressure.

On the day of admission, she developed dizziness while at work, associated with a sensation of impending syncope, tremulousness, and a racing heartbeat lasting several hours. She also reported sharp left-sided chest pain that was worse on deep inspiration. Her dizziness was predominantly experienced on standing and improved when sitting or resting. She described recurrent episodes of head pressure, reduced energy, and difficulty functioning normally because of her symptoms. There was no history of loss of consciousness, limb weakness, speech disturbance, dyspnea, hemoptysis, fever, or recent infection.

Her past medical history included iron deficiency anemia and migraine. She was a nonsmoker and independent in activities of daily living. There was no significant family history of rheumatological disease. She had experienced one miscarriage approximately five years previously. Her medications before admission were propranolol 10 mg twice daily and ferrous fumarate 210 mg daily.

On neurological examination, the patient was alert and fully oriented, with a Glasgow Coma Scale score of 15/15. Speech was normal, with no dysarthria or aphasia. Cranial nerve examination was unremarkable, with pupils equal and reactive to light, intact extraocular movements, no facial asymmetry, and intact visual fields. Motor examination demonstrated normal bulk and tone, with full power (5/5) in all four limbs and no pronator drift. Deep tendon reflexes were symmetrical and within normal limits, with flexor plantar responses bilaterally. Sensory examination was intact to light touch, pinprick, vibration, and proprioception. Coordination was preserved, with no dysmetria or dysdiadochokinesia. Gait was normal, including tandem gait where assessed. There was no focal neurological deficit.

Cardiovascular examination revealed carotid and subclavian bruits, with absent bilateral radial and brachial pulses and absent dorsalis pedis pulses. She had persistent hypotension with an orthostatic fall in blood pressure on standing.

The detailed examination findings are summarized in Table 1. These vascular abnormalities provided an important diagnostic clue in the context of her age and neurological symptoms.

**Table 1:** Clinical examination findings on admission.

Parameter	Finding
General appearance	Alert, orientated, Glasgow Coma Scale 15/15
Respiratory rate	17 breaths/minute
Oxygen saturation	97% on room air
Initial blood pressure	100/67 mmHg
Lowest recorded blood pressure	73/46 mmHg
Initial heart rate	57 beats/minute
Lying BP	85/62 mmHg
Standing BP	75/50 mmHg
Respiratory examination	Clear chest; no respiratory distress
Cardiovascular examination	Carotid and subclavian bruits ; no cardiac murmur
Radial pulses	Absent bilaterally
Brachial pulses	Absent bilaterally
Carotid pulses	Palpable bilaterally
Femoral pulses	Palpable bilaterally
Posterior tibial pulses	Palpable bilaterally
Dorsalis pedis pulses	Absent bilaterally
Neurological examination	Normal power and sensation; no focal neurological deficit
Eye movements	Intact
Visual fields	Intact
Cerebellar examination	No dysdiadochokinesia or ataxia
Nystagmus	No
Limb examination	No evidence of critical limb ischemia
Musculoskeletal examination	No synovitis
Skin examination	No rash or other vasculitic skin manifestations

Given the neurological symptoms, a CT scan of the head was performed. This demonstrated multiple supratentorial hypodensities involving the right frontal lobe and left corona radiata, without intracranial hemorrhage or definite evidence of acute evolving infarction (Figure 1). Following discussion with the stroke team, the imaging abnormalities were considered chronic, with no evidence of an acute stroke requiring acute intervention. Aspirin 75 mg daily was commenced, and MRI of the brain and CTA of the cerebral and carotid circulation were planned.

**Figure 1:**
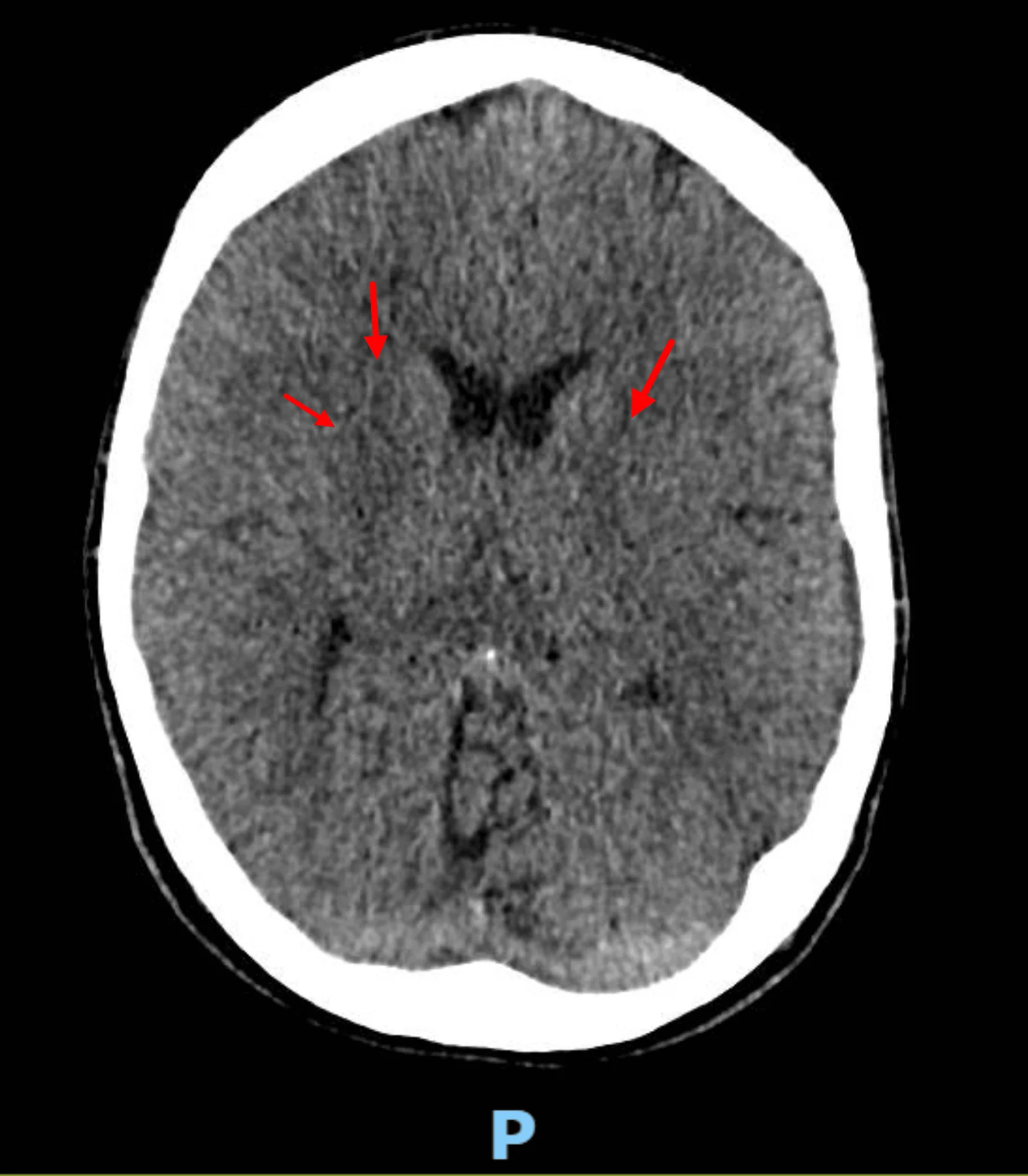
CT Head Demonstrating Chronic Cerebral Ischemic Changes (Red arrows) Axial Non-contrast computed tomography (CT) of the brain demonstrating multiple supratentorial hypodense areas involving the right frontal lobe and left corona radiata, consistent with chronic ischemic changes.

MRI of the brain subsequently demonstrated multiple chronic cerebral infarcts involving the bilateral centrum semiovale (Figure 2).

**Figure 2:**
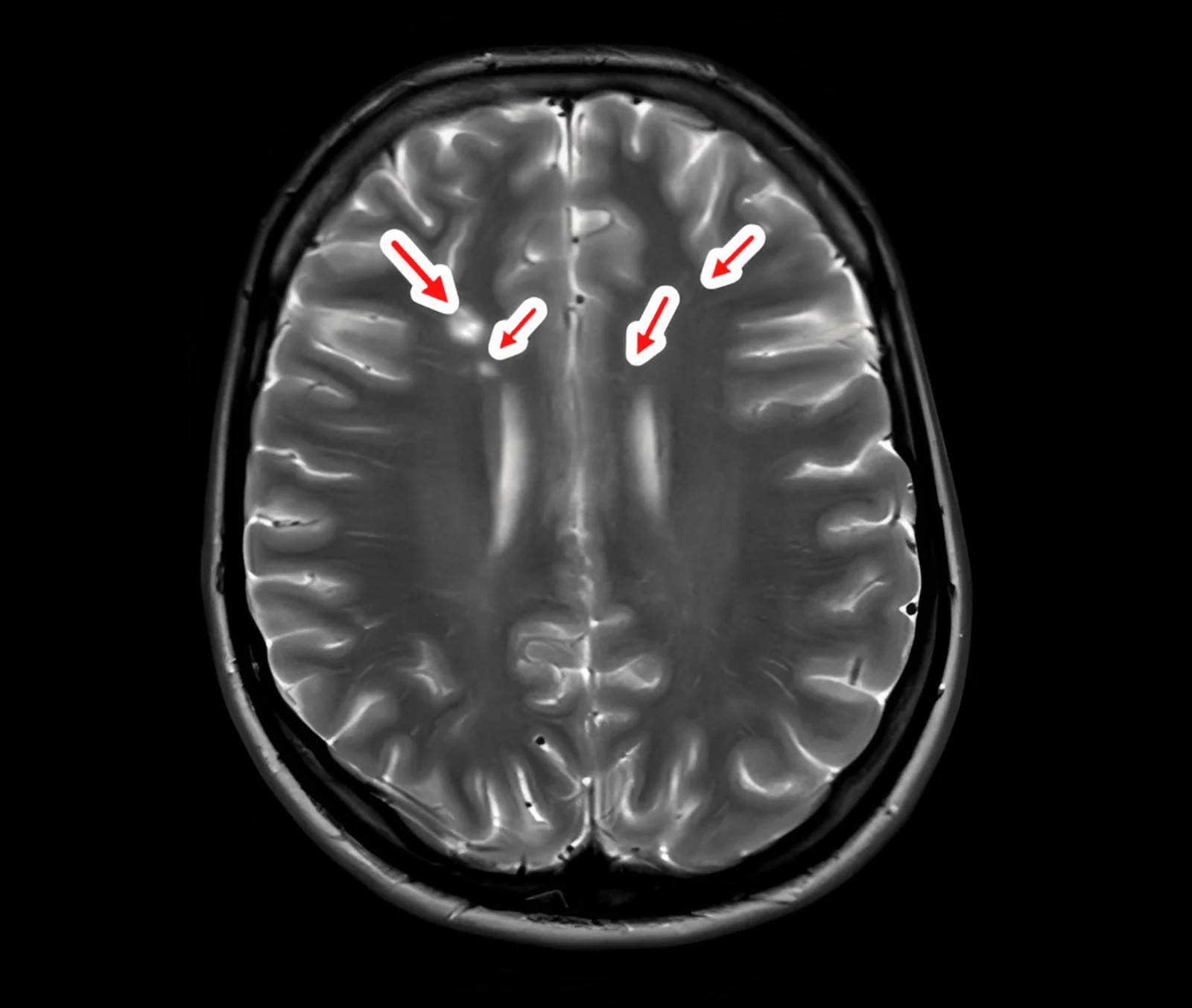
MRI brain demonstrating multiple chronic cerebral infarcts (red arrows). Axial T2-weighted magnetic resonance imaging (MRI) of the brain demonstrates multiple bilateral hyperintense lesions in the centrum semiovale (arrows), consistent with the chronic cerebral infarctions described in the radiological report.

CT angiography of the aortic arch, carotid arteries, and intracranial circulation subsequently demonstrated extensive arterial abnormalities. There was marked narrowing of the bilateral common carotid and subclavian arteries with associated wall thickening and peripheral calcification. The right internal carotid artery demonstrated critical stenosis at its origin, with severe wall thickening and persistent narrowing throughout its course. The left internal carotid artery demonstrated mild stenosis (Figure 3).

**Figure 3:**
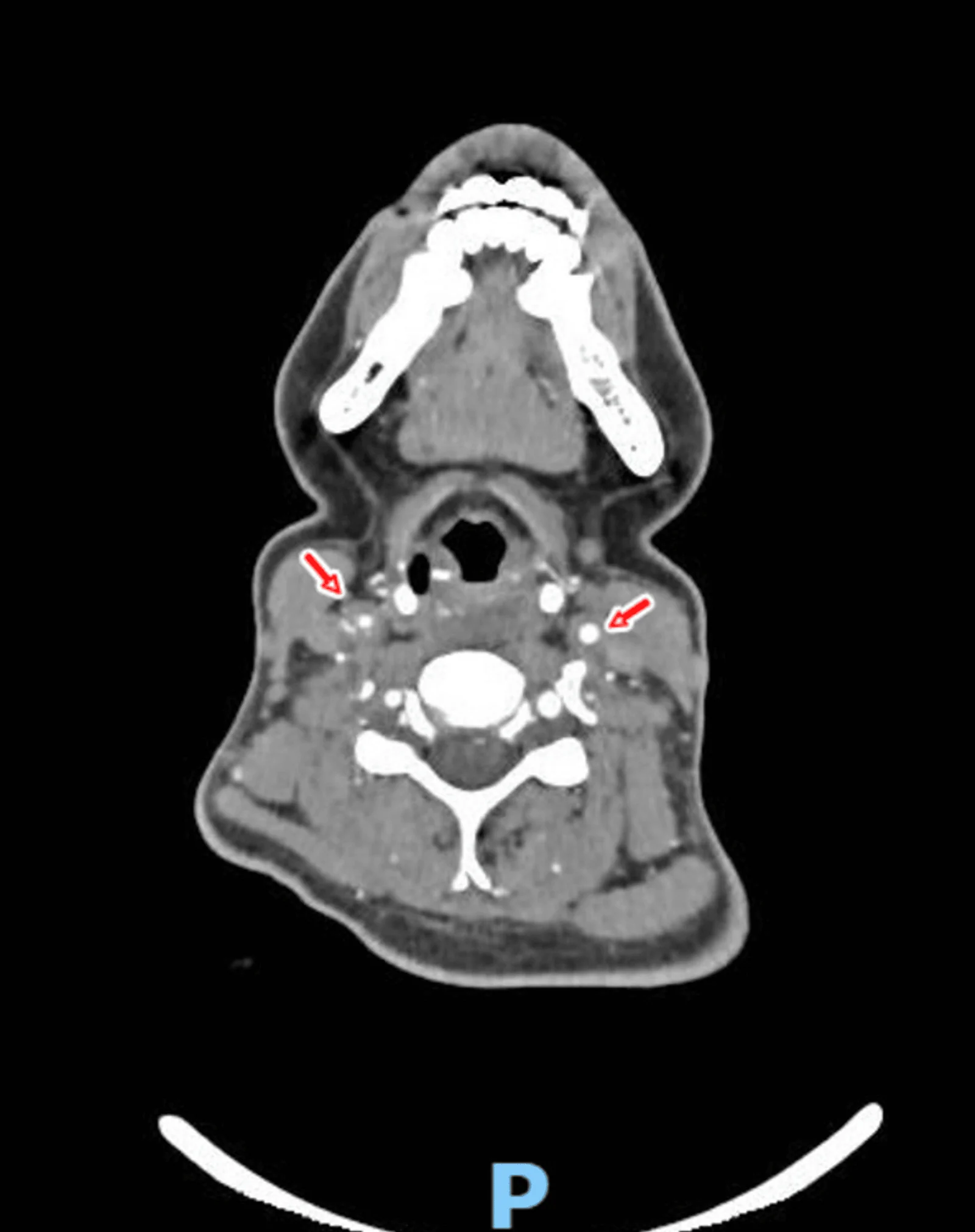
CT angiography demonstrating extensive large-vessel arterial disease (red arrows). Computed tomography angiography (CTA) demonstrates extensive bilateral common carotid stenosis with associated mural thickening. Critical stenosis is demonstrated at the origin of the right internal carotid artery, with mild stenosis of the left internal carotid artery.

Due to persistent hypotension and evidence of chronic cerebral infarctions, echocardiography was arranged and demonstrated preserved left ventricular systolic function with an ejection fraction >55%, with no significant cardiac abnormality identified (Figure 4). The key imaging findings obtained during the diagnostic evaluation are summarized in Table 2.

**Table 2:** Key imaging findings during the diagnostic evaluation. CT, computed tomography; MRI, magnetic resonance imaging; CTA, computed tomography angiography; Echo, echocardiography.

Investigation	Main findings
CT brain	Multiple supratentorial hypodensities involving the right frontal lobe and left corona radiata; no acute intracranial hemorrhage (Figure 1).
MRI brain	Multiple chronic cerebral infarcts involving the bilateral centrum semiovale; no acute infarction (Figure 2).
CT angiography	Extensive bilateral common carotid artery disease, critical stenosis of the right internal carotid artery, mild stenosis of the left internal carotid artery (Figure 3).
Echocardiography	Normal left ventricular systolic function with an ejection fraction >55% (Figure 4)

**Figure 4:**
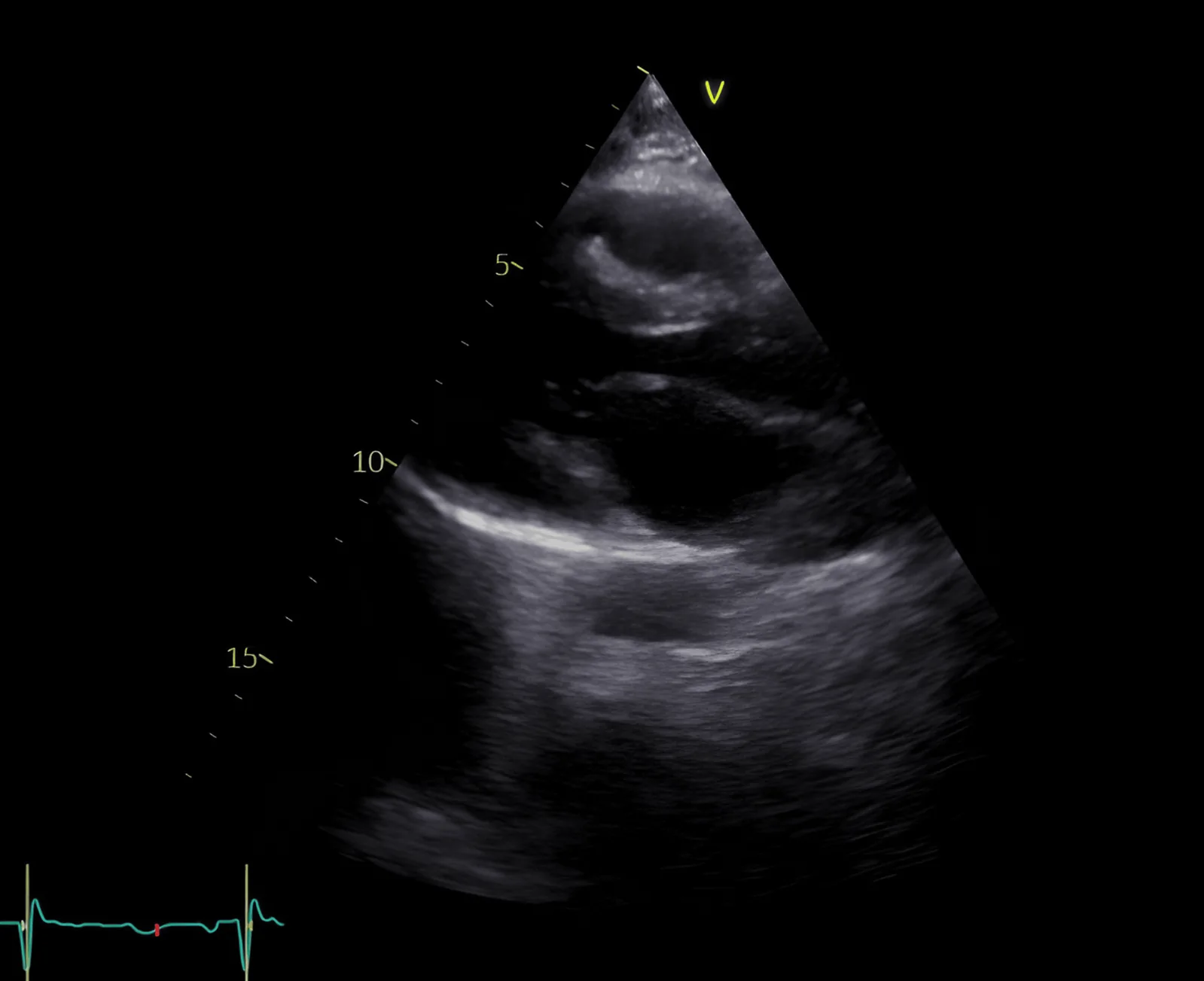
Echocardiography demonstrating preserved left ventricular systolic function. Transthoracic echocardiography demonstrates preserved left ventricular systolic function with an ejection fraction greater than 55%.

Laboratory investigations

Initial laboratory investigations were largely unremarkable apart from mildly elevated C-reactive protein (CRP) and erythrocyte sedimentation rate (ESR). Renal function, electrolytes, full blood count, cardiac enzymes, 9 AM cortisol, and thyroid function were within normal limits. Subsequent autoimmune, vasculitic, thrombophilia, and infectious investigations were largely unremarkable, apart from a weakly positive antinuclear antibody (ANA) and a positive lupus anticoagulant on one occasion. The complete relevant laboratory investigations are summarized in Table 3.

**Table 3:** Laboratory investigations during the diagnostic work-up. ALT: alanine aminotransferase; ALP: alkaline phosphatase; eGFR: estimated glomerular filtration rate; ESR: erythrocyte sedimentation rate; TSH: thyroid-stimulating hormone; ANA: antinuclear antibody; anti-dsDNA: anti-double-stranded DNA antibody; ANCA: antineutrophil cytoplasmic antibodies; IgG4: immunoglobulin G4; HIV: human immunodeficiency virus; TB: tuberculosis.

Investigation	Result	Reference range/interpretation
Hematology		
Hemoglobin	129 g/L	115–155 g/L
White blood cell count	8.7 × 10⁹/L	4.0–11.0 × 10⁹/L
Platelet count	235 × 10⁹/L	150–400 × 10⁹/L
Renal and metabolic profile		
Sodium	138 mmol/L	135–145 mmol/L
Potassium	4.5 mmol/L	3.5–5.0 mmol/L
Urea	3.2 mmol/L	2.5–7.8 mmol/L
Creatinine	45 µmol/L	45–90 µmol/L
eGFR	>90 mL/min/1.73 m²	>90 mL/min/1.73 m²
Liver profile		
ALT	44 U/L	<35 U/L
ALP	51 U/L	30–130 U/L
Total protein	84 g/L	60–80 g/L
Albumin	45 g/L	35–50 g/L
Globulin	39 g/L	20–35 g/L
Inflammatory markers		
C-reactive protein	19 mg/L → 9 mg/L	<5 mg/L
ESR	37 mm/hour	Elevated
Cardiac investigations		
High-sensitivity troponin I	<3 ng/L	<14 ng/L
Thyroid function		
TSH	1.25 mIU/L	0.4–4.0 mIU/L
Endocrine investigations		
9 AM cortisol	455 nmol/L	~140–690 nmol/L (morning reference range; laboratory dependent)
Autoimmune investigations		
ANA	Weakly positive	Previously reported
Anti-dsDNA	Unremarkable/negative	—
ANCA	Unremarkable/negative	—
Complement C3/C4	Unremarkable	—
IgG/IgG4	Unremarkable	—
Creatine kinase	Unremarkable	—
Extended myositis panel	Unremarkable	—
Antiphospholipid/thrombophilia investigations		
Lupus anticoagulant	Positive on one occasion	Abnormal
Anticardiolipin antibodies	Unremarkable	—
β2-glycoprotein antibodies	Unremarkable	—
Thrombophilia screen	Unremarkable	—
Infectious investigations		
Hepatitis B	Negative	—
Hepatitis C	Negative	—
HIV	Negative	—
QuantiFERON-TB Gold	Negative	—
Syphilis serology	Negative	—
Varicella IgG	Positive/immune	—

Ophthalmological assessment was performed because of her recurrent visual symptoms. Visual acuity was 6/9 in the right eye and 6/6 in the left eye. Both optic discs were healthy, with no evidence of retinal vasculitis. Cotton-wool spots and mild mid-peripheral retinal hemorrhages were identified in the right eye, findings considered compatible with mild ocular ischemic changes in the setting of significant carotid artery disease.

The patient was subsequently reviewed by the rheumatology team. Based on the overall clinical presentation, vascular examination, laboratory findings, and characteristic multiterritorial arterial abnormalities on CTA, the treating multidisciplinary team considered the clinical diagnosis to be TAK. The 2022 ACR/EULAR criteria were considered as a classification framework rather than as a stand-alone diagnostic tool. The patient had several features incorporated into these criteria, including age below 60 years, reduced upper-extremity pulses, arterial bruits, carotid arterial abnormality, and involvement of multiple arterial territories. The diagnosis was therefore based on the overall clinicoradiological assessment rather than on classification criteria alone.

The patient was treated with intravenous methylprednisolone, receiving a total of 2 g over three days, followed by oral prednisolone 40 mg daily with a planned gradual taper. Aspirin 75 mg daily was commenced.

Current ACR/Vasculitis Foundation recommendations generally favor the use of glucocorticoids in combination with a nonglucocorticoid immunosuppressive agent for active TAK. In this case, initial treatment focused on high-dose glucocorticoid therapy for presumed active vascular inflammation, together with aspirin. A nonglucocorticoid immunosuppressive agent was not initiated during the documented treatment period, as the patient showed clinical improvement with initial treatment.

Given the critical right internal carotid artery stenosis, the case was discussed with the vascular team. No immediate endovascular or surgical intervention was recommended, and the patient was managed medically with corticosteroid therapy and close multidisciplinary follow-up. No follow-up vascular imaging was available during the reported follow-up period to objectively determine whether the established arterial stenoses or arterial wall abnormalities had improved.

At the most recent documented follow-up, the patient reported marked improvement in dizziness, palpitations, visual disturbances, and constitutional symptoms following corticosteroid therapy. She was clinically stable and continued on a gradually reducing dose of oral prednisolone under rheumatology follow-up. These findings represent symptomatic improvement; they do not establish anatomical improvement of the underlying arterial stenoses. However, inflammatory markers were within normal limits, with a CRP of <4 mg/L and an ESR of 4 mm/hr, supporting the clinical improvement observed.

## Discussion

TAK is a chronic large-vessel vasculitis predominantly affecting the aorta and its major branches, most commonly in young women. Clinical manifestations depend on the vascular territories involved and may include constitutional symptoms, limb ischemia, visual disturbances, neurological symptoms, and cerebrovascular events [1-2]. This case is notable for an atypical presentation dominated by dizziness, presyncope, palpitations, visual disturbance, and hypotension, with subsequent identification of chronic cerebral infarcts and extensive extracranial arterial disease.

Neurological and vascular manifestations

Neurological manifestations of TAK may occur with stenotic or occlusive involvement of the carotid and vertebral arteries and may result from hemodynamic insufficiency, thromboembolism, or a combination of mechanisms [3]. In this patient, MRI demonstrated multiple chronic cerebral infarcts despite her young age and absence of significant conventional cardiovascular risk factors. Subsequent CTA demonstrated bilateral carotid stenosis with critical stenosis of the right internal carotid artery.

A particularly important diagnostic clue was the vascular examination, which demonstrated absent bilateral radial and brachial pulses, absent dorsalis pedis pulses, and carotid and subclavian bruits. These findings are among the clinical features incorporated into the 2022 ACR/EULAR classification criteria for TAK [1]. However, these criteria were developed for classification rather than as a stand-alone diagnostic test. In this case, the diagnosis was based on the integration of the clinical presentation, vascular examination, laboratory findings, and extensive multiterritorial arterial abnormalities demonstrated on CTA.

Role of vascular imaging

The diagnosis of TAK relies heavily on vascular imaging because the disease produces characteristic abnormalities involving the arterial wall and lumen. The 2023 EULAR imaging recommendations identify MRI as an important imaging modality in suspected TAK, while CTA and other imaging techniques can provide complementary information regarding vascular anatomy and disease extent [3].

In this case, CTA was particularly valuable because it demonstrated extensive multiterritorial arterial disease involving the bilateral common carotid, internal carotid, and vertebral arteries. The combination of extensive bilateral disease and arterial wall thickening favored a systemic large-vessel process rather than an isolated focal vascular abnormality.

The critical stenosis of the right internal carotid artery was especially important in explaining the patient's cerebral ischemic findings. The absence of a large-vessel intracranial occlusion and the presence of a patent circle of Willis further supported the importance of extracranial vascular disease in the overall clinical picture.

Inflammatory markers

The inflammatory response in this case was relatively modest despite extensive structural vascular disease. CRP was mildly elevated initially and subsequently decreased, while ESR remained moderately elevated.

Inflammatory markers are nonspecific in TAK and should be interpreted together with clinical findings and vascular imaging rather than in isolation [2-4]. This case illustrates the potential discordance between inflammatory markers and the anatomical extent of established arterial disease.

Importantly, the absence of markedly elevated inflammatory markers should not override compelling clinical and radiological evidence of large-vessel vasculitis.

Diagnostic considerations and differential diagnosis

The initial symptoms were nonspecific and could have suggested several alternative diagnoses, including a primary neurological disorder, cardiac arrhythmia, orthostatic intolerance, or migraine. The subsequent identification of chronic cerebral infarcts in a young woman prompted further investigation of the cerebral and extracranial circulation.

The differential diagnosis of extensive arterial stenotic disease in a young patient includes premature atherosclerotic disease, arterial dissection, fibromuscular dysplasia, antiphospholipid-associated vascular disease, and other forms of systemic vasculitis. In this case, the patient's age, absence of significant conventional cardiovascular risk factors, extensive bilateral and multiterritorial arterial involvement, arterial wall thickening, absent peripheral pulses, vascular bruits, and chronic cerebral ischemic lesions favored a large-vessel vasculitic process, with TAK considered the most likely diagnosis.

Although lupus anticoagulant was positive on one occasion, the remaining antiphospholipid and thrombophilia investigations were unremarkable. The overall clinical and radiological pattern was therefore considered more consistent with TAK than with an isolated thrombotic disorder.

Treatment and clinical response

Glucocorticoids remain an important component of treatment for active TAK. Current ACR/Vasculitis Foundation guidance recommends treatment with glucocorticoids together with a nonglucocorticoid immunosuppressive agent rather than glucocorticoids alone in patients with active TAK [4]. Treatment decisions should be individualized according to disease activity, vascular complications, comorbidities, and response to therapy [4-6].

In this case, the patient received intravenous methylprednisolone followed by oral prednisolone and aspirin. No nonglucocorticoid immunosuppressive agent was given during the period covered by this case report, with a clear plan from the rheumatology team to initiate such treatment at a subsequent follow-up visit.

Given the critical right internal carotid artery stenosis, the case was reviewed by the vascular team, and no immediate endovascular or surgical intervention was undertaken.

At the most recent documented follow-up, the patient reported marked improvement in dizziness, palpitations, visual disturbances, and constitutional symptoms. This symptomatic improvement occurred during corticosteroid treatment but should not be interpreted as evidence of anatomical regression of the arterial stenoses in the absence of follow-up vascular imaging.

Clinical implications

This case highlights that unexplained cerebral ischemia in a young patient should prompt consideration of underlying large-vessel disease, even when neurological symptoms are nonspecific and inflammatory markers are minimally elevated. A careful vascular examination, including peripheral pulse assessment, bilateral blood pressure measurement, and auscultation for bruits, may provide the key diagnostic clue. Any abnormal vascular findings or unexplained cerebral infarction should prompt vascular imaging rather than attribution to a benign neurological or autonomic disorder [1,3,5]. Early recognition of extensive arterial disease is essential to guide appropriate multidisciplinary management and reduce the risk of recurrent ischemic events.

Limitation 

This report is limited by its single-patient design and therefore cannot establish generalizability. No formal cerebral perfusion study was performed, so the precise mechanism of the chronic cerebral infarcts remains uncertain. Follow-up vascular imaging was unavailable to assess changes in arterial stenoses or vessel wall abnormalities. In addition, follow-up was primarily clinical, with reduced CRP but no serial ESR measurements or other objective vascular outcomes.

## Conclusions

TAK should be actively considered in young patients with unexplained cerebral ischemia, visual symptoms, dizziness, presyncope, or hypotension, especially in the absence of conventional vascular risk factors. This case demonstrates that severe extracranial arterial disease may present with subtle, nonspecific symptoms and may be overlooked when attention is focused solely on the neurological presentation. The key diagnostic message is simple: unexplained cerebral ischemia in a young patient warrants a vascular examination. Pulse asymmetry or absence, inter-arm blood pressure differences, and arterial bruits should immediately raise suspicion for large-vessel vasculitis and prompt comprehensive vascular imaging. Importantly, minimally elevated inflammatory markers do not exclude advanced arterial disease.

Early recognition of the vascular pattern is critical, as delayed diagnosis may result in recurrent cerebral ischemia, progressive arterial stenosis, and irreversible organ damage. In such patients, examining the pulses is not merely part of the physical examination; it may be the step that leads to the diagnosis.
